# Prediction of Flight Time Deviation for Lithuanian Airports Using Supervised Machine Learning Model

**DOI:** 10.1155/2020/8878681

**Published:** 2020-10-26

**Authors:** Pavel Stefanovič, Rokas Štrimaitis, Olga Kurasova

**Affiliations:** ^1^Faculty of Fundamental Science, Vilnius Gediminas Technical University, Saulėtekio al. 11, LT-10223 Vilnius, Lithuania; ^2^Institute of Data Science and Digital Technologies, Vilnius University, Akademijos str. 4, LT-08663 Vilnius, Lithuania

## Abstract

In the paper, the flight time deviation of Lithuania airports has been analyzed. The supervised machine learning model has been implemented to predict the interval of time delay deviation of new flights. The analysis has been made using seven algorithms: probabilistic neural network, multilayer perceptron, decision trees, random forest, tree ensemble, gradient boosted trees, and support vector machines. To find the best parameters which give the highest accuracy for each algorithm, the grid search has been used. To evaluate the quality of each algorithm, the five measures have been calculated: sensitivity/recall, precision, specificity, *F*-measure, and accuracy. All experimental investigation has been made using the newly collected dataset from Lithuania airports and weather information on departure/landing time. The departure flights and arrival flights have been investigated separately. To balance the dataset, the SMOTE technique is used. The research results showed that the highest accuracy is obtained using the tree model classifiers and the best algorithm of this type to predict is gradient boosted trees.

## 1. Introduction

For all airlines, flight time deviation from scheduled times brings financial, coordination, or technical difficulties. Passengers may also experience some difficulties or inconvenience when planning their time. The reasons for scheduled flight time deviation can be classified as primary and reactionary types. The reactionary type occurs when a delayed incoming flight has the knock-on effect of causing further delay, so the delay chain goes via all airports and becomes more complex. Over the past few years, a lot of new researches are made to predict reactionary type deviations. One of the effective proposed approach is multiagent based, the classification quality reaches to 80.7% [1]. The primary type is influenced by natural factors. Airports issues accounted for all delays over the past year mainly due to airport weather, airport capacity, maintenance or aircraft problems, air traffic capacity, control, and limitation [2]. In the research [3], the spatial analysis has been used to find the factors which influence the departure flight delay. The research results show that, in many cases, weather is one of the most important factors, so the weather-induced prediction methods are proposed in [4], also a lot depends on the size and activity of the analyzed airport. The small airport has not many flights in 24 h; therefore, the arrival time delay depends mostly on the reactionary type, and in the case of departure flight, deviation time is usually small. No matter what kind of delays is analyzed, all are described and presented in delay codes by the International Air Transport Association [5]. The codes can be used to different data mining-based approaches as a class value when the influence of the specific factors is analyzed [6]. Certainly, flight delay depends on so many factors; so to avoid at least basic problems, the airport structure optimization must be done [7].

The main aim of this paper is to find the best classification algorithm used in machine learning which can be suitable to adapt results for small airports delay analysis. The small countries usually got just a few active airports and no local flights. It is important to analyze such kind of dataset because usually only big airports are analyzed in the literature. The problem is that the bigger airports have to deal with more complex problems than the small airports have and the flight time deviation comes from various factors; so the solutions and results not always fit the small ones. Also, the bigger airports collected and presented in public flight information is much more detailed; moreover, the amount of data records is also much bigger (more airports in the country, bigger flight number in a day, and airport complexity). In the paper, the prediction of arrival and departure time is solved as a classification problem, where the main aim is to predict the interval of time deviation of new flights (new data). The newly collected dataset of Lithuania airport flight information and local area weather information are analyzed to predict possible flight time deviation from scheduled time [8]. There is no database where such kind of information would be freely accessed. In the paper, suggested data collecting model can be adapted for various countries airports analysis. At this moment, the dataset is not big but constantly updated; so in the future, the obtained results should be more accurate. The analysis is based on supervised machine learning model, where common algorithms have been used [9, 10] and results are compared, such as a probabilistic neural network, multilayer perceptron, decision trees, random forest, tree ensemble, gradient boosted trees, and support vector machines [11, 12]. To find the best parameters for each algorithm, the grid search is used. Some features of datasets used in delay analysis are not only numerical values but also categorical. Not all algorithms can usually deal with such kind of data; so the data encoding has to be done. There are significant different algorithms evaluations in the literature [13], but the widely used ones are accuracy, recall, precision, *F*-measure, etc. In the paper, the comparative analysis of algorithms results presented in Section 4.

## 2. Related Works

Nowadays, the flight delay analysis is one of the common research areas, which can be analyzed by various aspects and using different techniques. As it was mentioned before, the flight delay is influenced by various factors; one of the factors is the size of the airport. In the literature, usually, the big airports are analyzed and the problem is that the proposed methods are not suitable for small airports or all the countries' flight analysis. The suggested solution is complex and based on many more criteria. There are three main airports in Lithuania, and all flights from them are only going to the other countries in Europe, no local flights. The total number of flights per day is approximately 140. The literature review shows up that all most all similar size and activity airports, the main delay factors are weather, technical problems, and reactionary delay. In Zamkova's paper [14] the main goal was an examination of factors influencing flight delay at three most important international airports in the Czech Republic (Praha, Brno, Ostrava). 5777 flights are investigated in the overall period. The research results show up that delays caused by technical issues are most frequent in main airport Praha, same as delays caused by operational control. Also, it was proven that problems caused by a delayed departure from the previous destination (reactionary delays) are significantly more frequent in Brno and Ostrava which is probably caused by a low number of alternative available aircraft at these airports. The delays caused by technical defects or necessary maintenance take mostly a long time before the aircraft is ready for departure. Considering the research conclusions made in the Zamkova paper, in our research, the weather was selected as the main external factor for a possible flight delay. One more similar size airport analysis has been made in another research [15]. To detect flight delays in Egypt, the small dataset (512 records, 9 attributes) has been analyzed using various machine learning techniques (decision trees, PART Jrip, J48, etc.). It is not possible to compare the obtained results with results performed in our paper, because the parameters used for each classification algorithm are not presented, training and data preparation are missed, and also there is a quite big gap between analyzed dataset sizes. Also, instead of overall classification accuracy, *F*-measure and AUC/ROC have been evaluated. However, the best result is obtained using the PART classification.

The other research [16] is analyzing the problem of predicting flight departure delay at Porto (Portugal) airport. In the paper, the approach based on the so-called unimodal model is used to predict the delay. The unimodal model is implemented using neural networks and decision trees. The dataset consisted of 26189 regular commercial passenger flights performed during 2012, which was separated into training and testing subsets. The predictor variables were meteorological condition and flight information such as flight destination, airlines, and plane type. The experiment results show up that the arrival delay and the ground operation time are the most significant variables for departure delay prediction in Porto airport. An interesting thing is that neural networks and tree models had difficulty in distinguishing flights where departure delay falls in minutes from flights whose departure delay falls in minutes. To predict flight delay in Riga airport (Latvia), two groups have been investigated: delays related to airport procedure (maintenance, crew problems, ground handling, the number of delays (2686)) and other procedures (number of delays (6566)) such as weather and aircraft/airlines problems [17]. The deeper analysis has been made with each group separately after the authors split it into smaller groups. The result of research demonstrates the season character of a flight delay as well as the tight relation between ground handling services and aircraft flight delays.

The data mining and machine learning techniques have been used to predict the delay of American airlines (in the five most active airports of America) [18]. The dataset which was used in analysis consists of 97,360 samples with 12 features and 1 class label. Ten features are categorical and two numerical (departure/arrival times). All of them are directly related to information about the flight, and the weather or other factors is not analyzed. To evaluate obtained results the accuracy, recall, precision, and other measures have been used. The machine learning model has been trained using the gradient boosting trees and hyperparameter tuning it; the obtained accuracy is equal to 85.73%. A deeper literature analysis showed that the gradient boosted trees are one of the common classification algorithm used for flight delays when dataset size is big [19, 20]. The model accuracy using this algorithm is mostly always higher compared to other algorithms. The problem is that, in all cases, the results depend on various factors: dataset size, attributed number, attribute types, parameters used for each model, testing, etc. So, it is difficult to fully adapt research results, because usually just the overall model accuracy is presented, and other information is skipped. Also, as it was mentioned before, the smaller airport is rarely analyzed. In our paper, the grid search has been made to find and present the best parameters for each algorithm using a newly collected dataset. All data preparation, training, and testing steps are described.

## 3. Supervised Machine Learning Model

Machine learning (ML) and deep learning are subareas of artificial intelligence. The main aim of ML is the practice of using algorithms to analyze data, learn from results, and then make a determination or prediction about something in the world. For example, the machine learning model is trained to predict the weather according to the past weather information. There are many different types of machine learning algorithms [21], and they are always constantly updated or modified, but usually, they are grouped by learning style: supervised learning, unsupervised learning, and semisupervised learning. Also, ML models can be grouped on similarity in form or function such as classification, regression, clustering, prediction, and deep learning.

**Figure 1:**
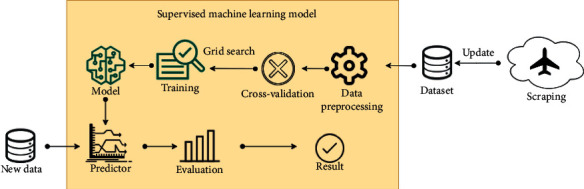
Supervised machine learning model.

In the paper, the supervised machine learning model has been used. The general scheme of the used model is presented in Figure 1. First of all, the model has been trained using a dataset according to the past flight information. The dataset is always updated, so the model always is retrained. In the data preprocessing node, the dataset have to be prepared correctly for each algorithm independently, because some of them have an issue with different variables type. Each algorithm is evaluated using cross-validation. Also, the random sampling is used. All analyzed algorithms are described in Section 3.1.

To find the best parameters for each method, the grid search has been implemented. The grid search allows us to change each parameter in initial parameter bounds to maximize the accuracy. The most common parameters have been varied. After training with the grid search, the model has been created. The new data are given to the classifier to find to which class they are assigned. In this research, as a new dataset, we used a randomly selected 20% of all datasets to find which algorithm gives the best results. Evaluation measures are described in Section 3.2.

### 3.1. Classification Algorithms

#### 3.1.1. Probabilistic Neural Network (PNN)

The probabilistic neural network is a feed-forward neural network architecture similar to MLP. It has 4 layers: input, pattern or hidden, summation, and output. The pattern layer is composed of trained neurons. Each trained node evaluates the input vector by Gaussian radial basis function which is just the Euclidean distance. The summation layer is composed of a class of nodes, where the sums are calculated from the corresponding trained class nodes. The outline layer simply identifies to which class the neuron belongs by finding class neuron with the highest sum. The method works only with numerical values.

#### 3.1.2. Multilayer Perceptron (MLP)

Multilayer perceptron is a forward neural network architecture made up of 3 types of layers: input, hidden, and output. Each neuron in the hidden layer is calculated by multiplying all the neurons from the previous layer by their weights and activated by an activation function. If the backpropagation algorithm is to be used, a nonlinear activation function is required. The error function is calculated in the output layer. If the result is not accurate enough, it is possible to perform a backpropagation algorithm that changes the neural weights and resubmits the input data to the multilayer perceptron.

#### 3.1.3. Decision Trees

Decision trees are a supervised learning algorithm that resembles a tree data structure. Tree construction works by first selecting the most appropriate attribute for a node (starting with the root node) and then splitting the data into subsets according to the selected attribute. Selection can be done using different criteria: entropy shows how random the information is; information gain shows how well a given attribute separates the training data; Gini index evaluates the splits in a dataset; gain ratio eliminates the branches with lots of small leaves. Tree construction ends when all leaves become pure matches 100% or 0% attribute of the class. The main problem is overfitting; each leaf of the tree has one value so the tree becomes adapted to training data and therefore shows poor results with new data. One solution to this problem is pruning: randomly divide the training data into two data sets: training and validation, and use the training data set to “grow” the tree, then use the validation data set to check how much the tree would become more accurate if you delete each node. Remove the node that provides the greatest improvement and repeat the process until the accuracy of the decision trees improves.

#### 3.1.4. Random Forest

The main difference between decision trees and random forest is that, in the training process, grows up not one, but *k* number of trees. Training data are randomly divided into *k* number of subsets, and each one grows a full tree (no pruning) using a subset of attributes. A prediction is done by submitting new data to all the trees in the random forest and new data belong to the class that most trees predicted.

#### 3.1.5. Tree Ensemble

It is one more decision tree-based algorithm where the main difference from previous methods is that the tree ensemble uses several learning algorithms to solve low bias and high variance problems of a single decision tree. Bagging and boosting are two most popular ensemble methods to achieve that. The main difference between bagging and random forest algorithms is that, in random forest, only a subset of features is selected at random out of the total and the best split feature from the subset is used to split each node in a tree, unlike in bagging where all features are considered for splitting a node.

#### 3.1.6. Gradient Boosted Trees

The learning process starts by creating a single root node tree. The root node represents an initial prediction for every training data. Usually, it is just the average value for regression or log-odds for classification. Then, a tree is built based on calculated residuals, the difference between training data values and prediction. Tree height is restricted. New predictions are made by calculating the output values of each tree leaf and scaling them with a learning rate. The algorithm repeats building trees until an additional tree fails to improve predictions (residuals gets too small) or the maximum number of trees is reached. The prediction process is performed by submitting new data to a created set of trees starting from the first tree to a single root node tree.

#### 3.1.7. Support Vector Machine (SVM)

A support vector machine is a supervised learning algorithm that finds optimal hyperplane dividing the dataset into two classes. The hyperplane is an (*n*−1)-dimensional subspace for an *n*-dimensional space, e.g., it is a line for 2-dimensional space and plane for 3-dimensional space. An optimal hyperplane is found by finding the largest distance from each hyperplane to the nearest point of any class. Linear and nonlinear functions (kernel) can be used for finding similarities between data points. Kernel functions calculate the relationships between every pair of points as if they are in the higher dimension without transforming data to the higher dimension (kernel trick).

### 3.2. Data Preprocessing and Evaluation Measures

Some of our analyzed algorithms can work just with numerical values, and categorical values are simply ignored. In the paper, the dataset consists of categorical and numerical values, and the full dataset description is presented in Section 4.1. Therefore, some variables of our dataset have to be converted to numerical. The two most used techniques are as follows: integer encoding (each category label is converted to unique integer number) and one-hot encoding (each category label is mapped to a binary vector). The primary research showed that there is no big difference between these techniques for our dataset at this moment; so the integer encoding has been used.

Five measures have been used to evaluate the quality of trained algorithms: sensitivity/recall, precision, specificity, *F*-measure, and accuracy. All these measures can be easily calculated from the confusion matrix [22]. The classifier result could be compared to actual result and summarized as follows: true positive (TPs), predicted positive and it is true, true negative (TN), predicted negative and it is true, false positive (FP), predicted positive and it is false; false negative (FN), predicted negative and it is false. Each measure formula is given in Table 1. First of all, each measure is calculated for separate classes. After that, the weighted-average is calculated to get the overall value of each measure.

To get accurate results for some algorithms (decision trees, MLP, etc.), the dataset has to be balanced; otherwise, the measures result will be misleading. For example, if we analyze 9 men and 1 woman dataset, the accuracy will be equal to 90%. So, if we give the new data to the model, the data item usually will be predicted as a man class. Balanced data are very rare in real life, so the data balancing methods have to be used. One of the widely used techniques is the Synthetic Minority Oversampling Technique (SMOTE) [23]. The SMOTE technique adjusts the class distribution by adding artificial rows that are created by extrapolating between a real object of a given class and one of its nearest neighbors (of the same class). After it, the technique picks a point along the line between these two objects and determines the attributes (cell values) of the new object based on this randomly chosen point.

## 4. Experimental Investigation

### 4.1. Dataset

**Figure 2:**
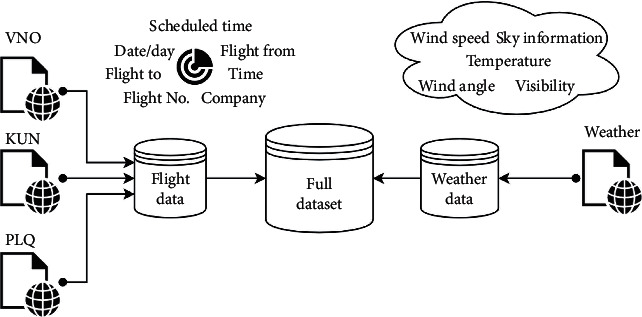
Data collecting model.

The flight time deviation in Lithuania airports has been analyzed. There are three main airports in Lithuania: Vilnius (VNO), Kaunas (KUN), and Palanga (PLQ). In our research, we used a web scraping technique to get all information about flights from official airport websites, and besides that, the weather condition has been collected too. The data collecting model is presented in Figure 2.

The activity of Lithuania airport is not high, so scrapping bot was taking the information every 15 minutes from 2019 October 26 until 2020 March 16. Just all unique flights have been stored. Because of the coronavirus situation over the all world, the Lithuania airports stopped all flights from and to Lithuania in 2020 March 16. The dataset is analyzed in this interval. The dataset is always updated and can be free accessed [8]. All datasets are separated into two subsets and analyzed independently: arrival flights and departure flights. In the paper, twelve-dimensional vectors have been analyzed, where  *X*_*m*_=(*x*_*m*1_, *x*_*m*2_,…, *x*_*m*12_) *m*=1,…, 7409 are arrival flights and  *Y*_*n*_=(*x*_*n*1_, *x*_*n*2_,…, *x*_*n*12_) *n*=1,…,  7057 are departure flights. The vectors features are as follows:

 
*x*_1_: flight date/day (numerical)
 
*x*_2_: flight no. (categorical)
 
*x*_3_: company (categorical)
 
*x*_4_: flight TO (categorical)
 
*x*_5_: flight FROM (categorical)
 
*x*_6_: temperature (numerical)
 
*x*_7_: sky information (categorical)
 
*x*_8_: wind speed (numerical)
 
*x*_9_: wind angle (numerical)
 
*x*_10_: visibility (numerical)
 
*x*_11_: scheduled time (numerical)
 
*x*_12_: classes (six values)

**Figure 3:**
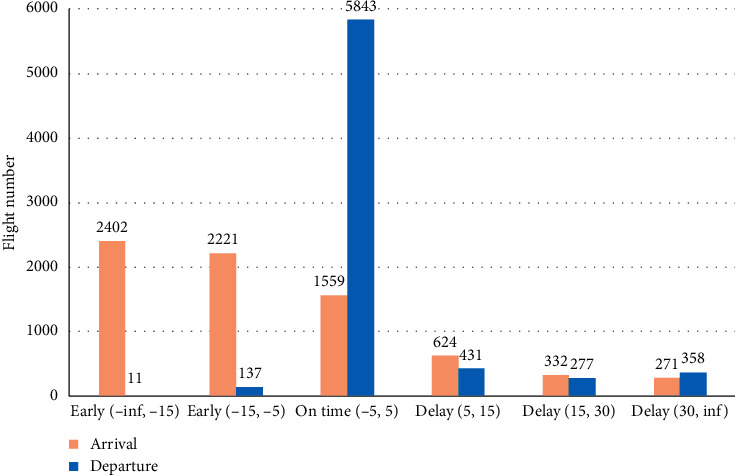
Flight time deviation in Lithuanian airports.

Various clustering algorithms can be helpful to determinate the data classes, such as *k*-means and self-organizing maps [24]. According to the Supporting European Aviation [2], the five-minute delay is not considered as a real delay, because the deviation is small. So, referred to other researches and EUROCONTROL recommendation, all data items have been assigned into one of such classes (intervals indicate time in minutes) according to past arrival and departure times: early (−inf, −15); early [−15, −5); on time [−5, 5]; delay (5, 15]; delay (15, 30]; delay (30, inf). As we can see in Figure 3, the dataset is not balanced. The majority of departure flights are on time and the rest of them have delayed. Vice versa the majority of arrival flight arrives too early.

As it was mention before, 20% of all datasets of each subset has been taken as a new data which was used in the training process. So, the arrival dataset is separated into the training data  *T*_*i*_^*A*^=(*x*_*i*1_, *x*_*i*2_,…, *x*_*i*12_)  *i*=1,…, 5927 and the new data *I*_*j*_^*A*^=(*x*_*j*1_, *x*_*j*2_,…, *x*_*j*12_) *i*=1,…, 1482. Exactly the same way the departure flights are separated into the following: the training data  *T*_*k*_^*D*^=(*x*_*k*1_, *x*_*k*2_,…, *x*_*k*12_)  *k*=1,…, 5645 and new data *I*_*l*_^*A*^=(*x*_*l*1_, *x*_*l*2_,…, *x*_*l*12_) *l*=1,…, 1412. The training dataset has been balanced using the SMOTE technique, so the training dataset size is increased to  *T*_*i*_^*A*^=(*x*_*i*1_, *x*_*i*2_,…, *x*_*i*12_) *i*=1,…, 17781 and *T*_*k*_^*D*^=(*x*_*k*1_, *x*_*k*2_,…, *x*_*k*12_)  *k*=1,…, 16935.

### 4.2. Experimental Results

All experiments were conducted under the same conditions. The same training dataset has been used for all algorithms. To avoid that some algorithms that cannot deal with the categorical data, the integer encoding has been used for training and new data. In the training of each model process, the grid search has been implemented. The most common parameters have been analyzed, and the bounds and the best-founded parameters have been presented in Table 2. The cross-validation has been made using 6 folds and random sampling with the same random seed; in this case, around 20% of training data have been used as a test dataset. 6 folds are an optimal number for such a size dataset. After the model is trained, the new data are given to the model to find to which class the data belong.

**Figure 4:**
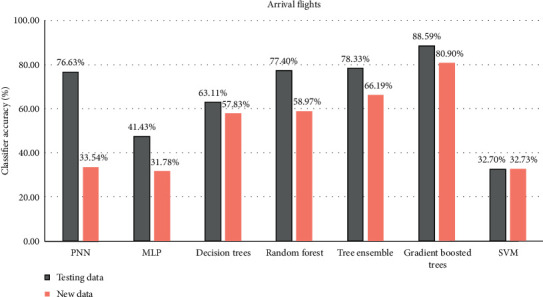
The algorithm comparison (overall accuracy) of arrival flights.

The classification accuracy of the two types of data is presented in Figure 4. The accuracy of the testing data is obtained after cross-validation. So, the results of the testing data represent the overall accuracy of each model. The new data are used just as input to the predictor to find to which class the data are assigned. It is important to note that the new data are not used in the training procedure; thus, they simulate the real situation of flights. We can see the best results are obtained using the gradient boosted tree technique (80.9%). The quite similar results are obtained using other tree model algorithms (∼60%). The probabilistic neural networks trained model accuracy is equal to 76.63%, but on the new data, it is just 33.54%. Multilayer perceptron and support vector machine give almost the same results on the new data; however, on the testing data, better one is MLP. All other evaluated measures values are presented in Table 3. As we can see, the support vector machine specificity is very small, the precision is low, and the sensitivity is quite higher compared to other algorithms. Also, the difference between *F*-measure and accuracy is around 15%. Meanwhile, all the measures of the gradient boosted trees are higher than other algorithms, so this method is the best option for such kind of data and the SVM is the worst.

**Figure 5:**
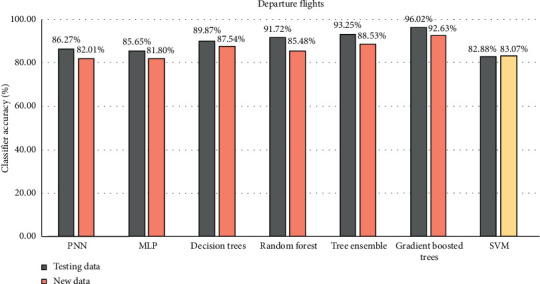
The algorithms comparison (overall accuracy) of departure flights.

The results using the departure dataset is quite similar to all algorithms (Figure 5), all of them are over 80%. As we can see in Figure 3, the majority of departure flights are always on time, variation is small, so it is the reason why results are so high. The highest accuracy is obtained using the gradient boosted trees (96.02%), the same as other evaluated measures (Table 4). The neural network-based algorithm (PNN and MLP) results are almost the same; just the specificity is lower compared to other algorithms. The smallest specificity is obtained using the support vector machine, but the overall accuracy is 82.88% on testing data and 83.07% on new data. It is hard to say which algorithm is the best using the departure flight data, but a slightly better algorithm is the gradient boosted trees. The other tree-based model (decision trees, random forest, and tree ensemble) results are similar. All of them are suitable to find time deviation on departure flight data with a high percentage.

## 5. Conclusions

In the paper, the prediction of flight time deviation has been performed. The model was based on the supervised machine learning and implemented in such a way: (1) past flights and weather dataset are separated to training and new data subsets; (2) the preprocessing of training and new datasets have been done; (3) each algorithm has been trained using grid search to find the best suitable parameters in the context of overall model accuracy; (4) the cross-validation has been used for evaluation; (5) the five measures have been used to evaluate created model; (6) the new data have been used to analyze and evaluate results. In the first step, all collected datasets are separated into two arrival and departure flights datasets. After, each dataset is separated into training and new subsets and analyzed separately. In the second step, the dataset balancing was performed using SMOTE technique and integer encoding has been used to make the same condition for each algorithm. All algorithms have been analyzed separately with grid search performance in the third step. The initial bound of parameters has been used to find the best parameters which obtain the highest accuracy. The cross-validation with 6 folds was used, and the five measures were evaluated to find the best algorithm to detect to which class the data belong. After all, the new dataset which was not used in any process has been given to the supervised machine learning model and results have been evaluated also.

The experimental investigation showed that the highest accuracy for arrival and departure subsets have been obtained using the gradient boosted trees (arrival, 88.59%; departure, 96.02%). The other measures compared to the rest algorithms were higher also. All tree-based models (decision trees, random forest, and tree ensemble) give quite similar results and accuracy average was around (arrival, 62%; departure, 96.02%), and all of them are quite fast. The probabilistic neural networks and multilayer perceptron (MLP) on the departure dataset obtain the high accuracy too (around 96.02%), but the MLP has problems with the arrival dataset (47%). The slow performance was using a support vector machine algorithm. The overall model accuracy on arrival flights is just equal to 32.70%, but on departure, flights are much higher and equal to 82.88%. The implemented supervised machine learning model showed that the best algorithm to find time deviation quick and with high accuracy is the gradient boosted trees. If the distribution of flight time varies a lot, the MLP and the SVM algorithms have to be avoided. The results will be more accurate after the dataset will be updated.

## Figures and Tables

**Table 1:** Evaluation measures for trained algorithms.

Measure	Formula
Sensitivity/recall	TP/(TP+FN)
Precision	TP/(TP+FP)
Specificity	TN/(TN+FP)
*F*-measure	(2 × TP)/(2 × TP+FP+FN)
Accuracy	(TP+TN)/(TP+TN+FP+FN)

**Table 2:** The grid search of classification algorithms.

Classification algorithm	Grid search					
	Parameter			Bounds	Best parameter arrival	Best parameter departure
Probabilistic neural network	Theta minus			From 0.1 to 0.9 by step 0.1	Theta minus: 0.1	Theta minus: 0.1
	Theta plus			From 0.1 to 0.9 by step 0.1	Theta plus: 0.5	Theta plus: 0.8
Multilayer perceptron	Iteration			From 10 to 500 by step 10	Iteration: 500	Iteration: 500
	Hidden layers number			From 1 to 4 by step 1	Layers: 3	Layers: 2
	Neurons number in the layer			From 10 to 100 by step 10	Neurons: 60	Neurons: 70
Decision trees	Split criterion			Gain ratio; Gini index	Split: Gini index	Split: Gini index
	Pruning method			Off: MDL	Pruning: MDL	Pruning: MDL
Random forest	Split criterion			Gain/gain ratio; Gini index	Split: Gini index	Split: Gini index
	Number of models			from 50 to 1000 by step 50	Models: 600	Models: 500
Tree ensemble	Split criterion			Gain/gain ratio; Gini index	Split: Gini index	Split: Gini index
	Number of models			From 50 to 1000 by step 50	Models: 450	Models: 150
	Attribute sampling (columns)			No sampling; square root; linear fraction; absolute value	Sampling: absolute	Sampling: absolute
	Fraction to learn single model			ON/OFF	Value: 9	value: 5
	Fraction to learn single model			From 0.1 to 1 by step 0.1	Fraction: OFF	Fraction: OFF
Gradient boosted trees	Tree depth			From 1 to 15 by step 1	Tree depth: 12	Tree depth: 9
	Number of models			From 50 to 1000 by step 50	Models: 300	Models: 300
	Learning rate			From 0.1 to 1 by step 0.1	Learning rate: 0.2	Learning rate: 0.2
	Attribute sampling (columns)			No sampling; square root; linear fraction; absolute value	Sampling: absolute Value: 10	Sampling: absolute Value: 7
	Fraction to learn single model			ON/OFF	Fraction: ON	Fraction: ON
	Fraction to learn single model			From 0.1 to 1 by step 0.1	Fraction: 1	Fraction: 1
Support vector machines	Overlapping penalty			From 1 to 10 by step 1	Overlapping: 3 Kernel RBF: 0.2	Overlapping: 2 Kernel RBF: 0.4
	Kernel	Polynomial	Power	From 1 to 10 by step 0.5		
			Bias	From 1 to 10 by step 0.5		
			Gamma	From 1 to 10 by step 0.5		
		Hypertangent	Kappa	From 0.1 to 1 by step 0.1		
			Delta	From 0.1 to 1 by step 0.1		
		RBF	Sigma	From 0.1 to 1 by step 0.1		

**Table 3:** The arrival flight estimation results (expressed as a percentage).

Algorithm	Dataset	Measures (%)				
		Sensitivity/recall	Precision	Specificity	*F* measure	Accuracy
Probabilistic neural network	Testing	78.87	76.63	90.28	77.23	76.63
	New data	36.56	33.21	74.17	34.55	33.54
Multilayer perceptron	Testing	56.73	47.43	73.01	50.77	47.43
	New data	39.45	32.15	66.87	35.17	31.78
Decision trees	Testing	66.56	63.11	85.78	64.42	63.11
	New data	61.12	57.84	83.55	59.12	57.83
Random forest	Testing	81.27	77.40	89.82	78.40	77.40
	New data	64.20	58.82	82.77	60.41	58.97
Tree ensemble	Testing	81.00	78.33	90.93	79.04	78.33
	New data	69.72	66.15	86.47	67.14	66.19
Gradient boosted trees	Testing	89.45	88.59	96.00	88.85	88.59
	New data	81.98	80.75	93.28	81.09	80.90
Support vector machine	Testing	91.98	32.70	6.47	46.41	32.70
	New data	96.42	32.01	4.26	47.50	32.73

**Table 4:** Departure flights' estimation results (expressed as a percentage).

Algorithms	Dataset	Measures (%)				
		Sensitivity/recall	Precision	Specificity	*F* measure	Accuracy
Probabilistic neural network	Testing	96.46	86.27	26.84	90.14	86.27
	New data	94.00	80.77	10.38	86.66	82.01
Multilayer perceptron	Testing	90.90	85.65	48.15	87.86	85.65
	New data	88.04	80.76	33.52	84.07	81.80
Decision trees	Testing	91.78	89.87	71.47	90.65	89.87
	New data	89.35	87.68	69.63	88.37	87.54
Random forest	Testing	94.94	91.72	65.66	92.82	91.72
	New data	89.97	84.68	46.51	86.60	85.48
Tree ensemble	Testing	94.68	93.25	76.96	93.74	93.25
	New data	90.02	88.48	70.72	89.11	88.53
Gradient boosted trees	Testing	96.62	96.02	87.33	96.22	96.02
	New data	93.35	92.06	79.18	92.52	92.63
Support vector machine	Testing	99.86	82.88	0.70	90.53	82.88
	New data	99.82	83.04	2.17	90.63	83.07

## Data Availability

The dataset used in this paper is available at https://www.kaggle.com/pavelstefanovi/lithuanian-airports-flight-dataset.

## References

[1] Guleria Y., Cai Q., Alam S., Li L. (2019). A multi-agent approach for reactionary delay prediction of flights. *IEEE Access*.

[2] EUROCONTROL: https://www.eurocontrol.int/our-data 2020

[3] Cheng S., Zhang Y., Hao S., Liu R., Luo X., Luo Q. (2019). Study of flight departure delay and causal factor using spatial analysis. *Journal of Advanced Transportation*.

[4] Choi S., Kim Y. J., Briceno S., Mavris D. Prediction of weather-induced airline delays based on machine learning algorithms.

[5] The international air Transport association. https://www.eurocontrol.int/publication/all-causes-delay-and-cancellations-air-transport-europe-q3-2019 2020.

[6] Zámková M., Prokop M., Stolín R. (2017). Factors influencing flight delays of a European airline. *Acta Universitatis Agriculturae et Silviculturae Mendelianae Brunensis*.

[7] Gilbo E. P. (1993). Airport capacity: representation, estimation, optimization. *IEEE Transactions on Control Systems Technology*.

[8] Lithuania airports flight dataset: https://www.kaggle.com/pavelstefanovi/lithuanian-airports-flight-dataset 2020

[9] Kotsiantis S. B. (2007). Supervised machine learning: a review of classification techniques. *Informatica*.

[10] Sagar S., Nikam A. (2015). Comparative study of classification techniques in data mining algorithms. *Oriental Journal of Computer Science & Technology*.

[11] Ding Y. Predicting flight delay based on multiple linear regression.

[12] Ujjwala U., Richariya P. (2016). Naive Baye’s classification algorithm in prediction of flight delays using MR. *International Journal of Innovative Research in Technology*.

[13] Hossin M., Sulaiman M. N. (2015). A review on evaluation metrics for data classification evaluations. *International Journal of Data Mining & Knowledge Management Process*.

[14] Zámková M., Prokop M. (2015). The evaluation of factors influencing flights delay at Czech international airports. *Acta Universitatis Agriculturae et Silviculturae Mendelianae Brunensis*.

[15] Altabbakh S., Mohamed H. M., EI Z. H. (2018). Machine learning techniques for analysis of Egyptian flight delay. *International Journal of Data Mining & Knowledge Management Process*.

[16] Alonso H., Loureiro A. Predicting flight departure delay at Porto airport: a preliminary study.

[17] Aloma I., Tolujew J., Medvedevs A., Kabashkin I., Yatskiv I., Prentkovskis O. (2017). Analysis of riga international airport flight delays. *Reliability and Statistics in Transportation and Communication. RelStat*.

[18] Chakrabarty N. A. Data mining approach to flight arrival delay prediction for American airlines. 2019 9th annual information technology.

[19] Liu F., Sun J., Liu M., Yang J., Gui G. Generalized flight delay prediction method using gradient boosting decision tree.

[20] Chakrabarty N., Kundu T., Dandapat S., Sarkar A., Kole D. Flight arrival delay prediction using gradient boosting classifier.

[21] Kubat M. (2015). *An Introduction to Machine Learning*.

[22] Ting K. M. (2011). *Encyclopedia of Machine Learning*.

[23] Chawla N. V., Bowyer K. W., Hall L. O., Kegelmeyer W. P. (2002). SMOTE: synthetic minority over-sampling technique. *Journal of Artificial Intelligence Research*.

[24] Stefanovič P., Kurasova O., Štrimaitis R. (2019). The n-grams based text similarity detection approach using self-organizing maps and similarity measures. *Applied sciences*.

